# Evidence of weak genetic structure and recent gene flow between *Bactrocera dorsalis* s.s. and *B. papayae*, across Southern Thailand and West Malaysia, supporting a single target pest for SIT applications

**DOI:** 10.1186/1471-2156-15-70

**Published:** 2014-06-14

**Authors:** Nidchaya Aketarawong, Siriwan Isasawin, Sujinda Thanaphum

**Affiliations:** 1 Department of Biotechnology, Faculty of Science, Mahidol University, Rama VI Road, Bangkok 10400, Thailand

**Keywords:** *Bactrocera dorsalis* complex, Area-wide integrated pest management, Sterile insect technique, Population genetics, Gene flow

## Abstract

**Background:**

*Bactrocera dorsalis* s.s. (Hendel) and *B. papayae* Drew & Hancock, are invasive pests belonging to the *B. dorsalis* complex. Their species status, based on morphology, is sometimes arguable. Consequently, the existence of cryptic species and/or population isolation may decrease the effectiveness of the sterile insect technique (SIT) due to an unknown degree of sexual isolation between released sterile flies and wild counterparts. To evaluate the genetic relationship and current demography in wild populations for guiding the application of area-wide integrated pest management using SIT, seven microsatellite-derived markers from *B. dorsalis* s.s. and another five from *B. papayae* were used for surveying intra- and inter-specific variation, population structure, and recent migration among sympatric and allopatric populations of the two morphological forms across Southern Thailand and West Malaysia.

**Results:**

Basic genetic variations were not significantly different among forms, populations, and geographical areas (*P* > 0.05). Nonetheless, two sets of microsatellite markers showed significantly different levels of polymorphisms. Genetic differentiation between intra- and inter-specific differences was significant, but low. Seventeen populations revealed three hypothetical genetic clusters (*K* = 3) regardless of forms and geographical areas. The genetic structure of sympatric populations slightly changed during the different years of collection. Recent gene flow (*m* ≥ 0.10) was frequently detected whether samples were sympatric or allopatric. Ninety-five of 379 individuals distributed across the given area were designated as recent migrants or of admixed ancestry. As a consequence of substantial migration, no significant correlation between genetic and geographic distances was detected (*R*^2^ = 0.056, *P* = 0.650).

**Conclusions:**

According to the 12 microsatellite variations, weak population structure and recent gene flow suggest that there is no status for cryptic species between *B. dorsalis* s.s. and *B. papayae* forms in Southern Thailand and West Malaysia. Both forms can be treated as a single target pest for the SIT program in an area-wide sense. Additionally, the result of species identification based on molecular data and morphological character are not congruent. The use of independent, multiple approaches in the characterization of the target population may ensure the effectiveness and feasibility of SIT-based control in the target area.

## Background

The sterile insect technique (SIT) is a powerful biological control method. It relies on mating between sterile insects (biological control agent) and their wild counterparts in order to reduce reproductive potential. SIT is effective because it is a species-specific approach and one of the most environmentally friendly solutions to insect pest management. However, there are several steps: mass-rearing, sex-separation for male-only release, sterilization, marking, and mass-releasing [1]. SIT is widely included in area-wide pest control programs against high-profile key pests of economic and biomedical importance. Examples of successful insect pest control programs using SIT include the eradication of the New World screwworm fly from the United States, Mexico, and Libya, the Mediterranean fruit fly from the northern part of Chile and the southern part of Peru, and the melon fly from Japan [2]. To maximize SIT effectiveness, the mating success between the sterile males and wild females should be enhanced. Consequently, these females will lay sterile eggs, leading to population disruption, which reduces overall pest damage.

Problems such as potential sexual isolation due to the existence of cryptic species and/or population isolation must be investigated before SIT application [3]. Cryptic species comprise two or more nominal species, which are sometimes morphologically indistinguishable. They may be recently or deeply diverged in sympatric or allopatric locales [4]. Resolution of cryptic species is needed for the management of many types of field operations related to pest control, conservation, and infectious diseases [4,5]. Ignoring cryptic species potentially undermines the SIT program due to ambiguous mating compatibility [4]. Mating incompatibility may contribute to the pattern of population isolation. An unsuccessful case of eradication using the SIT-based approach occurred for the New World screwworm [3] in Jamaica. Population isolation was present after several years of application, indicating the existence of a sexually cryptic species or a mating incompatibility between released sterile and wild insects [6].

The *Bactrocera dorsalis* species complex, a large group of nominal tephritid fruit flies, is of interest to scientists in several fields of study. Regarding morphological characters and host preferences, the *B. dorsalis* complex consists of almost 100 similar species [7,8]. Four members, *B. dorsalis* sensu sticto (Hendel), *B. papayae* Drew & Hancock (known before as *B. dorsalis* sensu lato), *B. carambolae* Drew & Hancock, and *B. philippinensis* Drew & Hancock, are key invasive agricultural pests in South East Asia [7-11]. *B. dorsalis* s.s. and *B. papayae* were reported to be parapatric species. *B. dorsalis* s.s. is distributed from the far north to the northern part of the Malay Peninsula while the species range of the other extends throughout the northern Malay Peninsula all across to the southern end, overlapping the range of *B. dorsalis* s.s around the Isthmus of Kra [7-11]. Allopatrically, *B. carambolae* and *B. philippinensis* are separately found in the Indonesian archipelago and the Philippines, respectively. Focusing on only Southern Thailand and the Malay Peninsula areas, the delimited distribution of *B. dorsalis* s.s. in the Malay Peninsula is uncertain [7-10]. According to only morphological forms, *B. dorsalis* s.s. and *B. papayae* are still classified as distinct species within the complex, although species limit has often been ambiguous [12,13]. On the other hand, the classification of *B. papayae* has recently been revised to be synonymized with *B. philippinensis*[8]. Nowadays, research into the species complex status has arrived at the question regarding the actual number of true economic species in such a complex, which is important for pest quarantine and management, as well as international trade [11].

Non-morphological characters (molecular markers, mating behaviour, and male pheromones), combined with morphological characters, have provided better resolution in the analysis of species limitation within several cryptic species [4]. Similarly, the biological status of members of *B. dorsalis* complex - *B. dorsalis* s.s., *B. papayae*, and *B. phillipinensis* - has recently been analyzed using independent multiple approaches such as studying several genes of mitochondrial DNA, microsatellite markers, and mating competitiveness [13-16]. This evidence suggests that they are the same entity. Among several molecular traits, the genetic diversity of microsatellite DNA could be a powerful tool for the study of species complex [17]. Strong inferences with less bias can be made when the microsatellite markers derived from both of the cryptic members are used. In addition, population samples are frequently collected throughout their geographical distribution in order to observe cline.

Microsatellites are short tandem repeats (2–5 bp) that are widely distributed in the genome and are inherited in Mendelian fashion. Such markers usually present high levels of intra- and inter-specific variations. Recently, several microsatellite DNA markers in tephritid fruit flies were developed and amplified across species (e.g., *Bactrocera papayae*[18], *B. musae*[19], *B. oleae*[20], *Rhagoletis cerasi*[21], *Anastrepha obligua*[22], *Ceratitis capitata*[23,24], and *C. rosa*[25]). Some of them have been subsequently used for elucidating the population genetic structure of the species complex [26,27]. However, microsatellite DNA markers isolated from one species transferred to other species may provide low genetic diversity due to the clone selection procedure (known as ascertainment bias) [28-30].

The current research aims to use microsatellite markers for generating high-resolution population genetic data from morphological forms *- B. dorsalis* s.s. and *B. papayae* - spanning from the top of Southern Thailand to the tip of the Malay Peninsula (West Malaysia). Population sampling was carried out using intervals of no more than 200 km. Sympatric populations of the two forms were also collected. To avoid the negative effect from an ascertainment bias of microsatellite DNA variation, we used microsatellite DNA markers derived from both *B. dorsalis* s.s. [31] and *B. papayae*[18] for surveying the intra- and inter-specific variation, genetic structure, and population dynamics. Furthermore, these data were subsequently used for evaluating the feasibility of SIT application in given areas. The degree of interbreeding between the two morphological forms can also be inferred.

## Methods

### Fruit fly samples and genomic DNA extraction

**Figure 1:**
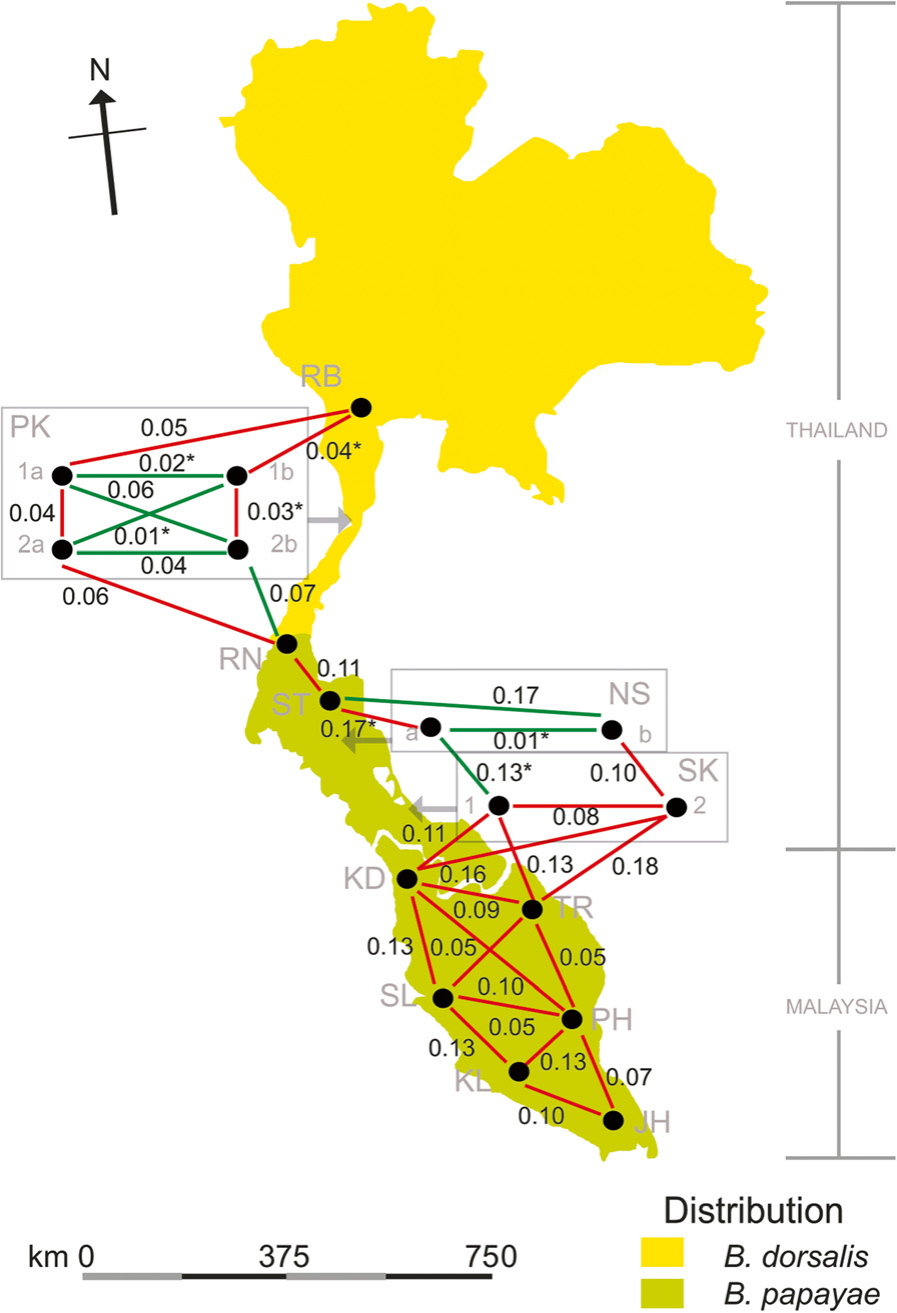
**Sampling sites of *B. dorsalis *s.s. and *B. papayae *in this study.** Seventeen fruit fly populations were collected from the top of Southern Thailand to the end of the Malay Peninsula (West Malaysia). Information for each sample is described in Table 1. The Isthmus of Kra (connecting zone between yellow and green areas) is the putative transition zone between *Bactrocera dorsalis* s.s. (yellow area) and *B. papayae* (green area) distribution [7,11]. Intra- and inter-specific differences (*F*_ST_) among nearby populations are reported. The red line represents intra-specific difference (pairwise *F*_ST_ between populations with the same morphological form) while the green line represents inter-specific difference (pairwise *F*_ST_ between populations with different morphological forms). An asterisk (*) indicates a non-significant *F*_ST_ value. Rectangular boxes detail the designated locations (by arrow) where either sympatric or temporal populations were collected.

Males of *Bactrocera dorsalis* s.s. and *B. papayae* were collected using methyl eugenol traps. Seventeen fruit fly populations corresponding to 12 areas - spanning from the top of Southern Thailand to the tip of the Malay Peninsula (West Malaysia) - were collected at no more than 200 km intervals (Table 1, Figure 1). The identification of fruit fly forms was carried out based on published descriptions by Drew and Hancock [7]. Three-hundred and seventy-nine individuals, with conforming morphological characters, were subsequently genotyped and analyzed.

**Table 1:** **Sample collection of *Bactrocera dorsalis *** **s.s. and *B. papayae *** **forms from Southern Thailand to West Malaysia**

**Assumed species according to morphological form**[7]	**Country**	**Sampling site**	**Sampling code**	**Sampling size**	**Collection time**	**Coordinates**
*B. dorsalis* s.s.	Thailand	Ratchaburi	RB	30	2008	13°25′N 99°43′E
		Prachub Kirikan	PK1a	30	2008	12°14′N 99°52′E
			PK2a	30	2010	
		Ranong	RN	27	2008	9°55′N 98°35′E
		Surat Thani	ST	24	2008	8°50′N 99°11′E
		Nakhon Sri Thammarat	NSa	3	2011	8°09′N 99°49′E
*B. papayae*	Thailand	Prachub Kirikan	PK1b	8	2008	12°14′N 99°52′E
			PK2b	31	2010	
		Nakhon Sri Thammarat	NSb	12	2011	8°09′N 99°49′E
		Songkhla	SK1	16	2010	7°06′N 100°25′E
			SK2	25	2011	
	Malaysia	Kedah	KD	21	2010	5°41′N 100°38′E
		Terengganu	TR	27	2010	5°38′N 102°29′E
		Selangor	SL	26	2010	3°58′N 102°26′E
		Pahaug	PH	25	2010	3°43′N 100°56′E
		Kuala Lumpur	KL	27	2010	3°04′N 101°45′E
		Johor	JH	17	2010	2°01′N 103°18′E

Sampling sites consisted of the following locales: Ratchaburi (RB), Prachub Kirikan (PK), Ranong (RN), Surat Thani (ST), Nakhon Sri Thammarat (NS), Songkhla (SK), Kedah (KD), Terengganu (TR), Selangor (SL), Pahang (PH), Kuala Lumpur (KL), and Johor (JH) (Table 1). Notably, populations of *B. dorsalis* s.s. and *B. papayae* forms were found to be sympatric in Prachub Kirikan and Nakhon Sri Thammarat. In addition, temporal populations were collected from Prachub Kirikan and Songkhla.

Fruit fly samples were preserved in 95% ethanol and kept at −20°C until use. Total genomic DNA was individually extracted from each fly [31].

### Microsatellite amplification and genotyping

Sets of seven (*Bd*1, *Bd*9, *Bd*15, *Bd*19, *Bd*39, *Bd*42, and *Bd*85B) and five (*Bp*58, *Bp*73, *Bp*125, *Bp*173, and *Bp*181) microsatellite loci - previously isolated and characterized from *B. dorsalis* s.s. [31] and *B. papayae*[18], respectively - were used (Additional file 1: Table S1). Microsatellite DNA amplifications were set up in a 15- μl volume reaction containing 100 ng of genomic DNA, 1 × buffer, 2.5 mM MgCl_2_, 25 μM dNTPs, 0.5 U *Taq* polymerase (Vivantis) and 5 μM of each primer. PCRs were performed using a thermal cycler Flexcycler (AnalytikJena, Germany) using the following conditions: 5 min at 94°C, 29 cycles of 30 s at 94°C, 90 s at *T*_a_ of each primer pair (Additional file 1: Table S1) and 90 s at 72°C, and an additional 5 min of elongation at 72°C at the end of the process. Electrophoresis and allele scoring were determined in 6% or 12% (w/v) non-denaturing polyacrylamide gel in 1xTBE buffer at 800 V for 6 or 10 hrs, respectively, stained with 0.5 mg/l ethidium bromide [32] and photographed under UV light. PCR product size was measured with a 25 bp DNA ladder (Promega, USA). For each locus analysis, samples with no PCR product were declared null allele (missing data), if two PCR attempts had been carried out.

### Data analysis

Test of the ascertainment bias hypothesis: Basic genetic variability (i.e., variance in PCR product size range (*V*_m_), number of allele (*n*_a_), and expected heterozygosity (*H*_E_)) was estimated for each population. However, in this study, we used the effective number of allele (*n*_e_), instead of *n*_a_ because it is less sensitive to sample sizes and rare alleles [33]. The *n*_e_ was calculated as *n*_e_ = 1/(1-*H*_E_). *H*_E_ was calculated as *H*_E_ = 1- (Σ*q*_i_^2^), where *q*_i_ is the frequency of the *i*^th^ allele in the population. *V*_m_ and *n*_e_ were in an approximately normal distribution. Each of the two measurements was analyzed by ANOVA using these factors: microsatellite DNA loci derived-species, *B. dorsalis* s.s. and *B. papayae*, populations, and countries. Conversely, *H*_E_ was transformed to 1/*H*_E_ and subsequently tested with the non-parametric Mann–Whitney U test with the following factors: microsatellite DNA loci derived-species, *B. dorsalis* s.s. and *B. papayae*, and countries. Only the population factor was tested with a Kruskal-Wallis test. All statistical analyzes were performed using PASW statistics software v18.0 (ó SPSS). The significant level is below 0.05 (*P* < 0.05).

Genetic diversity: The parameters for population genetic analyzes, i.e., *n*_a_, *n*_e_, number and frequency of private alleles (*n*_p_ and *A*_p_, respectively), observed heterozygosity (*H*_O_), *H*_E_, and inbreeding coefficient (*F*_IS_), were estimated using GENALEX v.6.5 [34]. In addition, rarefaction allele and private allele richness (*R*_s_ and *R*_p_, respectively) were estimated using HP_Rare [35]. The frequency of null alleles (*A*_n_) was estimated following Brookfield [36]. Deviation from Hardy-Weinberg equilibrium and linkage disequilibrium was determined using GENEPOP v.4 software [37] together with their critical levels after the sequential Bonferroni test [38].

Population genetic structure: We evaluated genetic structure using four different approaches: (i) measuring genetic differentiation (*F*_ST_) among populations, (ii) Bayesian model-based clustering, (iii) the Principle Coordinate Analysis (PCoA), and (iv) a hierarchical AMOVA. The first approach quantified genetic differentiation, pairwise *F*_ST_, among 17 populations using MICROSATELLITE ANALYSER (MSA) [39]. Estimation of *F*_ST_ values and their statistical significance was done for 10,000 permutations.

The Bayesian approach was used to determine genetically distinct groups (or clusters) using the program STRUCTURE v.2.3.1 [40,41]. To identify the number of genetic clusters within a location, in particular with low sample sizes from some populations, the no admixture model was initially studied [5]. This model hypothesizes that each individual belongs to one cluster. STRUCTURE was run with the Infer Lambda option set at the number of clusters (*K*) equal to one and five independent iterations. Subsequently, the mean of five lambda values was set with the option of correlated allele frequency for all additional runs. The other parameters were set at default: different values of *F*_ST_ for different subpopulations, prior *F*_ST_ mean of 0.01, and a standard deviation of 0.05. We ran STRUCTURE using *K* = 1 to *K* = 17. For each *K* value, five iterations were performed with the condition of a burn-in period of 100,000 iterations followed by a run of 500,000 Markov Chain Monte Carlo (MCMC) repetitions. The optimal *K* value was estimated by examining the Ln *P*(*X/K*) output from STRUCTURE [40] and calculating the Δ*K* statistic [42].

Also, to recognize the potential admixture between genetic clusters, the admixture model was additionally run with the same parameters in the no admixture analysis. The location prior option (or sample assignment prior in this case) was included in order to allow for revealing weak population structure, but does not find structure when none is present. Moreover, it is able to ignore prior assignment when a correlation between the clusters and sampling locations is not observed [43]. All data were summarized using CLUMPP v.1.1.2b [44] and visualized through DISTRUCT v.1.1 [45].

The PCoA using GENALEX v.6.5 [34], performed on genetic distance, was used to display genetic divergence among the individual fruit flies in multidimensional space using allele frequency data. A plot of the first three principal coordinates was constructed using the subprogram MOD3D in NTSYS-pc v.2.1 [46].

A hierarchical spatial AMOVA was performed using ARLEQUIN v.3.1.1., with 1,000 permutations [47]. Populations were grouped corresponding to three major criteria, i.e., morphological form, geographical area, and population genetic structure, to test genetic homogeneity in different hierarchies.

Analysis of genetic distances: The MSA program [39] was also used for estimating genetic distance based on Nei’s genetic distance [48] and the proportion of shared alleles [49]. The programs NEIGHBOR and CONSENSE in the PHYLIP package [50] were used to reconstruct the neighbor-joining trees after 1,000 bootstraps of the original data. Subsequently, the TreeView program was used for phylogenetic tree visualization [51].

Demographic inferences: Information regarding demography was investigated through three analyzes: (i) assignment test, (ii) population contraction/expansion, and (iii) isolation by distance (IBD). GENECLASS v.2.0 [52] was run to estimate the probability of each individual belonging to its own population, the probability of being an immigrant from each of the other populations, and the probability of being a migrant to other populations. A standard criterion, the Bayesian method [53], with enabled probability computation and Monte–Carlo re-sampling, following the simulation algorithm for population assignment was used [54]. An arbitrary threshold probability value of 0.100 was determined after simulating 10,000 genotypes for each population. BOTTLENECK v.1.2.02 [55] was run to detect the signal of demographic expansion/contraction in each population. The heterozygosity excess was tested under two proposed models of microsatellite mutation: the stepwise mutation model (SMM) and the two-phased mutation model (TPM). The latter model comprised 90% single-step and 10% multiple-step mutation. To avoid the effect of too few individuals and loci tested per population, Wilcoxon signed-rank test was performed [55]. Finally, the IBD analysis was performed using the ISOLDE option in the GENEPOP package to find the correlation between genetic and geographic distances [39].

## Results

### Testing ascertainment bias on genetic variability in *B. dorsalis* s.s. and *B. papayae* forms

Statistical analyzes were performed to compare different grouping factors and basic parameters for estimating genetic variations. No significant differences between ‘*B. dorsalis* s.s. and *B. papayae*’ for *V*_m_ (*F*_1,32_ = 0.735, *P* = 0.398) or *n*_e_ (*F*_1,32_ = 0.019, *P* = 0.891) were observed. Likewise, all of those parameters were not significantly different among populations: *V*_m_ (*F*_16,17_ = 0.336, *P* = 0.983) and *n*_e_ (*F*_16,17_ = 0.320, *P* = 0.986). The genetic variations were not significantly different between samples collected from Thailand and Malaysia: *V*_m_ (*F*_1,32_ = 0.482, *P* = 0.493) and *n*_e_ (*F*_1,32_ = 0.001, *P* = 0.975). The species-specific-derived microsatellite DNA primer factor was significantly different for all parameters: *V*_m_ (*F*_1,32_ = 31.634, *P* < 0.001) and *n*_e_ (*F*_1,32_ = 64.917, *P* < 0.001). The *B. dorsalis* s.s.-derived microsatellite DNA loci were more variable than the *B. papayae*-derived loci. However, there were no significant relationships between ‘species-specific-derived microsatellite DNA primer’ and ‘*B. dorsalis* s.s. and *B. papayae*’: *V*_m_ (*F*_1, 1_ = 0.002, *P* = 0.964) and *n*_e_ (*F*_1, 1_ = 1.387, *P* = 0.248). For the mean expected heterozygosity (*H*_E_), the ‘species-specific-derived microsatellite DNA primer’ factor was significantly different (*U* = 18.500, *P* < 0.001), but not for these factors: population, species, and country.

### Genetic variability

Hardy-Weinberg exact tests were performed for 12 microsatellite loci. After sequential Bonferroni correction [38], 55 out of the 204 population by locus comparisons significantly departed from Hardy-Weinberg expectations. However, all deviations were not concentrated in any population or at any locus. No significant linkage disequilibrium was detected between genotypes at the 12 loci.

All microsatellite loci reveal different levels of polymorphism among populations as summarized in Table 2. Within each population, the mean of *R*_s_ values is 3.23 ± 0.64, with the highest value observed in RN population (3.51). The *n*_p_ values are detected in all samples, except for three populations (ST, NSa, and PK1b). They range from one (KD and SL) to seven (SK2) with a low average frequency (0.02 and 0.06, respectively). JH shows the highest *A*_p_ value (0.13) at a low number (*n*_p_ = 3). Likewise, using the rarefaction approach, the mean *R*_*p*_ value is 0.15 ± 0.19, with the highest value detected in SK2 (0.28). The average *H*_E_ values in 17 populations vary from 0.54 (NSa) to 0.75 (RN). All corresponding average *H*_O_ values are lower, ranging from 0.37 (SK2) to 0.60 (PK1b and SK1). The presence of null alleles at a moderate frequency (0.10 – 0.20) could contribute to the observed heterozygote deficiencies in all populations. The values for the inbreeding coefficient (*F*_IS_: ranging from −0.04 to 0.42) and number of rare alleles (*n*_r_: ranging from 0 to 41) were taken from the given populations.

**Table 2:** **Summary of genetic variability among populations of *Bactrocera dorsalis *** **s.s. and *B. papayae *** **forms**

**Assumed species according to morphological form**[7]	**Code**	***n***_**a**_	***n***_**e**_	***n***_***r***_	***A***_***r***_	***n***_**p**_	***A***_**p**_	***R***_**S**_	***R***_***p***_	***H***_**O**_	***H***_**E**_	***A***_***n***_	***F***_**IS**_
*B. dorsalis* s.s.	RB	6.92	4.02	28	0.023	6	0.04	3.43 ± 0.68	0.27 ± 0.31	0.42	0.73	0.18	0.42
	PK1a	6.83	3.76	28	0.026	3	0.02	3.42 ± 0.48	0.14 ± 0.12	0.50	0.73	0.15	0.31
	PK2a	6.00	3.44	20	0.033	3	0.02	3.31 ± 0.49	0.09 ± 0.10	0.41	0.70	0.17	0.40
	RN	6.75	3.96	28	0.026	6	0.02	3.51 ± 0.56	0.14 ± 0.09	0.59	0.75	0.13	0.19
	ST	4.58	2.96	13	0.033	0	0.00	2.94 ± 0.44	0.13 ± 0.22	0.51	0.65	0.15	0.21
	NSa	2.50	2.16	0	0.000	0	0.00	2.58 ± 0.90	0.01 ± 0.05	0.44	0.54	0.20	−0.04
*B. papayae*	PK1b	4.50	3.28	0	0.000	0	0.00	3.35 ± 0.62	0.10 ± 0.13	0.60	0.70	0.10	0.06
	PK2b	7.17	3.39	41	0.029	3	0.02	3.28 ± 0.45	0.16 ± 0.09	0.44	0.70	0.15	0.35
	NSb	5.17	3.26	14	0.042	4	0.05	3.23 ± 0.68	0.15 ± 0.16	0.48	0.68	0.13	0.24
	SK1	5.17	3.20	11	0.031	2	0.05	3.21 ± 0.61	0.15 ± 0.16	0.60	0.70	0.16	0.06
	SK2	6.67	3.37	31	0.031	7	0.06	3.24 ± 0.78	0.28 ± 0.38	0.37	0.66	0.18	0.38
	KD	5.50	3.37	17	0.040	1	0.02	3.17 ± 0.59	0.13 ± 0.11	0.45	0.68	0.14	0.32
	TR	5.67	3.42	17	0.026	2	0.02	3.20 ± 0.67	0.10 ± 0.08	0.41	0.67	0.17	0.35
	SL	6.42	3.46	26	0.025	1	0.02	3.27 ± 0.71	0.16 ± 0.10	0.43	0.68	0.14	0.34
	PH	6.33	3.67	22	0.029	2	0.02	3.41 ± 0.60	0.16 ± 0.15	0.49	0.71	0.15	0.32
	KL	5.67	3.25	20	0.028	2	0.06	3.15 ± 0.61	0.14 ± 0.26	0.50	0.68	0.12	0.25
	JH	5.42	3.62	12	0.030	3	0.13	3.19 ± 0.63	0.22 ± 0.26	0.41	0.71	0.17	0.40

*n*_a_, mean number of alleles; *n*_e_, mean effective number of alleles, 1/(1-*H*_E_); *n*_r_, mean number of rare alleles (allele frequency ≤ 0.05); *A*_r_, mean frequency of rare alleles; *R*_S_, allele richness; *R*_*p*_, private allele richness; *n*_p_, number of private alleles; *A*_p_, mean frequency of private alleles; *H*_O_, mean observed heterozygosity; *H*_E_, mean expected heterozygosity; *A*_*n*_, mean frequency of null alleles, [(*H*_E_ – *H*_O_)/(*H*_E_ + 1)] [36]; *F*_IS_, mean inbreeding coefficient.

### Population structure

Genetic differentiation among populations was measured by the fixation index (*F*_ST_) (Table 3, Figure 1). Pairwise *F*_ST_ values are significantly different from zero, ranging from 0.011 (between NSa and NSb) to 0.183 (between ST and SK2). Non-significant differentiation was observed, especially when samples were paired with small sample size populations, (*N* < 10) for PK1b and NSa. All sympatric population pairs illustrate very low (PK2a and PK2b = 0.044) or non-significant differentiation (PK1a and PK1b = 0.020, and NSa and NSb = 0.011). Similarly, samples from the same locale, but collected a few years apart, reveal low pairwise *F*_ST_ values, i.e., PK1a and PK2a (0.037), PK1b and PK2b (0.031), and SK1 and SK2 (0.083). The degree of genetic differentiation (pairwise *F*_ST_) between the two morphological forms (ranging from 0.014 to 0.183) is comparable to the values observed among the same ‘morphopopulations’ (ranging from 0.021 to 0.148 in the *B. dorsalis* s.s. form, and from 0.019 to 0.181 in the *B. papayae* form).

**Table 3:** **Pairwise *F***_**ST **_**values among the 17 populations of *B. dorsalis *** **s.s. and *B. papayae *** **forms**

**Code**	**RB**	**PK1a**	**PK2a**	**RN**	**ST**	**NSa**	**PK1b**	**PK2b**	**NSb**	**SK1**	**SK2**	**KD**	**TR**	**SL**	**PH**	**KL**
PK1a	0.051															
PK2a	0.056	0.037														
RN	0.049	0.021^ns^	0.055													
ST	0.148	0.113	0.109	0.105												
NSa	0.064^ns^	0.098^ns^	0.081^ns^	0.069^ns^	0.172^ns^											
PK1b	0.035^ns^	0.020^ns^	0.014^ns^	0.038^ns^	0.126	0.042^ns^										
PK2b	0.063	0.056	0.044	0.070	0.126	0.072^ns^	0.031^ns^									
NSb	0.056	0.078	0.082	0.076	0.167	0.011^ns^	0.038^ns^	0.052								
SK1	0.079	0.085	0.051	0.081	0.101	0.126^ns^	0.064^ns^	0.042	0.103							
SK2	0.094	0.137	0.123	0.130	0.183	0.181	0.142	0.119	0.101	0.083						
KD	0.095	0.062	0.082	0.051	0.146	0.140^ns^	0.077	0.106	0.093	0.114	0.162					
TR	0.111	0.046	0.064	0.069	0.083	0.108^ns^	0.064	0.098	0.122	0.127	0.179	0.085				
SL	0.112	0.085	0.059	0.086	0.089	0.148	0.083	0.112	0.138	0.118	0.181	0.130	0.052			
PH	0.110	0.071	0.066	0.074	0.046	0.126^ns^	0.078	0.099	0.120	0.100	0.155	0.097	0.045	0.042		
KL	0.107	0.091	0.087	0.077	0.163	0.108^ns^	0.069	0.097	0.080	0.119	0.158	0.109	0.117	0.132	0.125	
JH	0.075	0.045	0.050	0.046	0.114	0.096^ns^	0.019 ^ns^	0.055	0.082	0.056^ns^	0.124	0.063	0.061	0.077	0.070	0.095

‘ns’ indicates values are not significantly different from zero.

**Figure 2:**
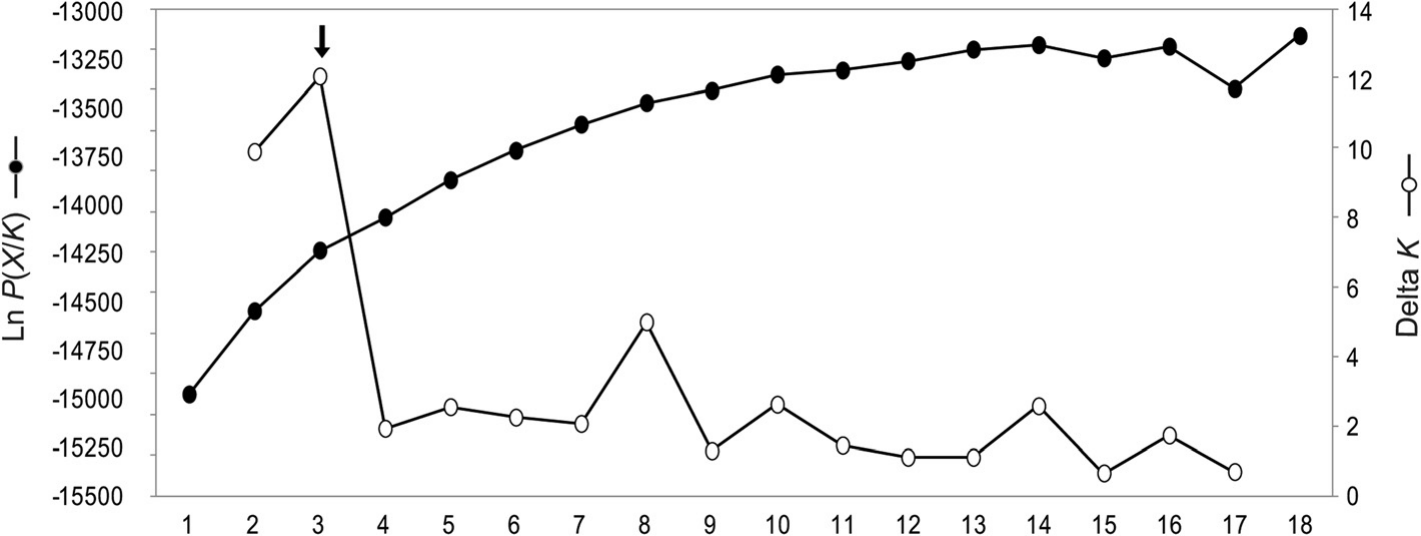
**Log-likelihood probability (Ln *P*(*X*/*K*)) and the Delta *K *values of data.** Three is indicated to be the most likely number of hypothetical genetic cluster (*K*) using admixture model.

**Figure 3:**
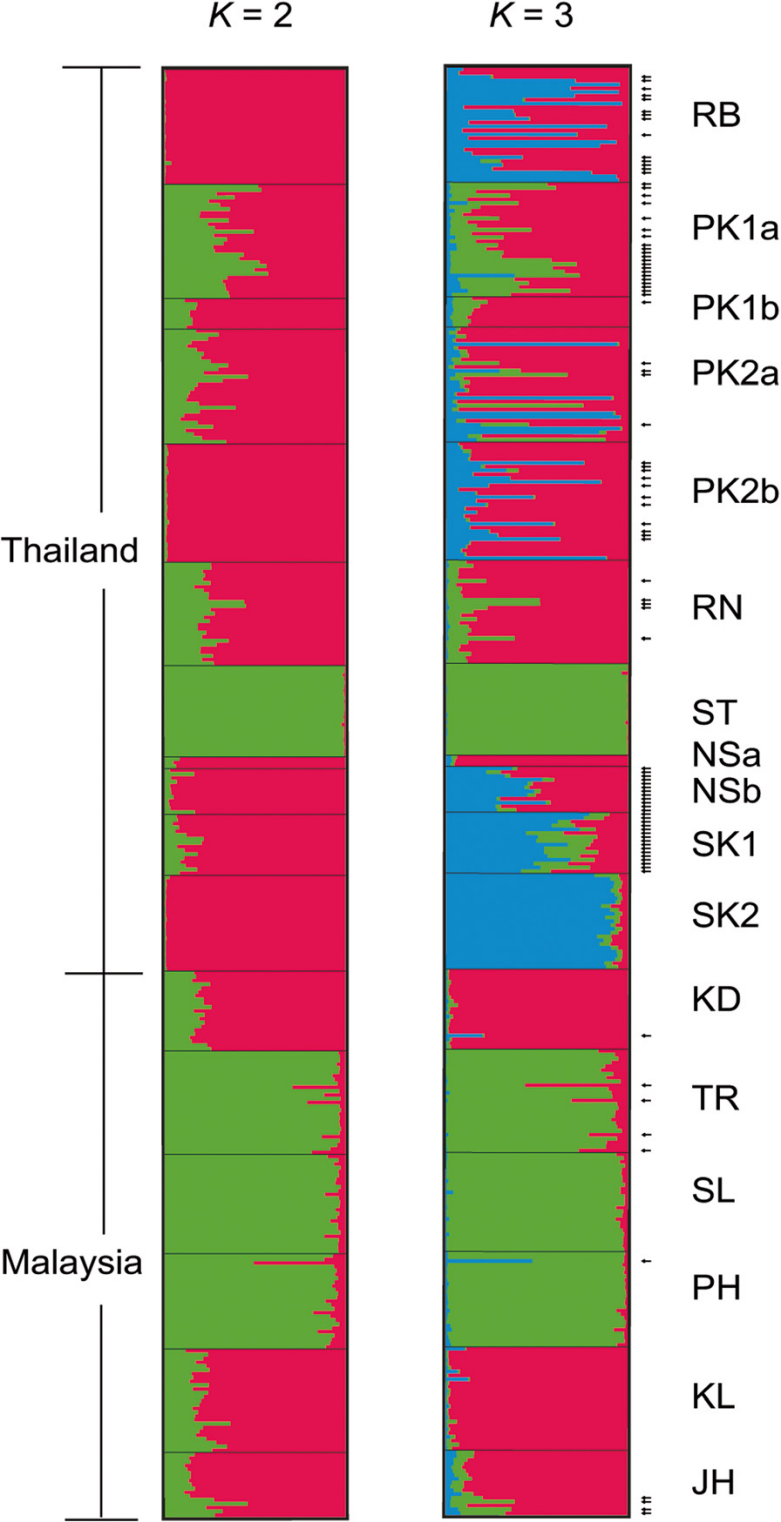
**STRUCTURE analysis (admixture model) of 379 individuals of *B. dorsalis *s.s. and *B. papayae *assigned to two and three genetic clusters (*K *= 2 and *K *= 3, respectively).** Each horizontal stripe represents an individual. Each color represents the proportion of membership with regard to the each hypothetical genetic cluster. Five replicates were combined into one figure using CLUMPP [44] and DISTRUCT [45]. Arrows indicate admixed individuals with a mean proportion of genetic cluster (*Q*) between 0.200 to 0.800, most present in samples from Southern Thailand.

**Figure 4:**
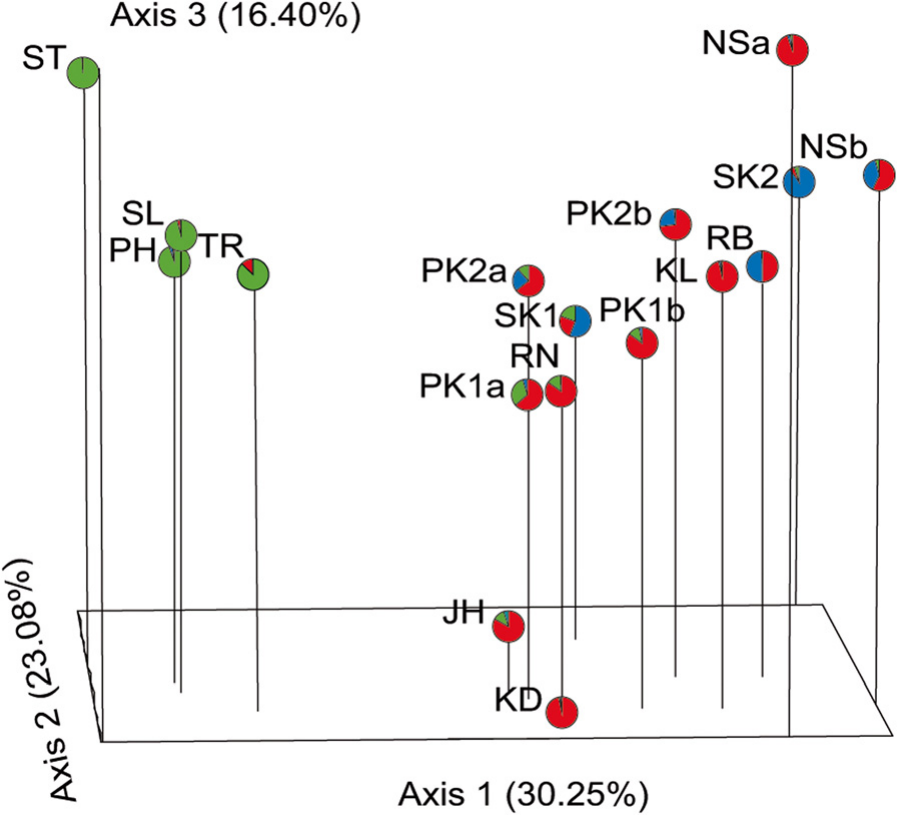
**Principle Coordinate Analysis (PCoA) in three-dimensional plot.** The planes of the first three principal coordinates explain 30.25%, 23.08%, and 16.40% of total genetic variation, respectively. The pie graph represents the average co-ancestry distribution of 379 individuals in three hypothetical clusters (*K* = 3).

To identify the number of hypothetical genetic clusters, STRUCTURE analyzes were run using both no admixture and admixture models. The Δ*K* value indicates that *K* equals three (*K* = 3) is the optimal number of hypothetical genetic clusters in both no admixture and admixture models (Figure 2). However, members in each genetic cluster are not related to any morphological forms. At *K* = 3, nine populations (i.e., RB, PK1a, PK2a, PK2b, RN, NSb, SK1, TR, and JH) reveal an admixed structure (Figure 3). Populations ST, TR, SL, and PH are separated from the rest with a major proportion of co-ancestry (*Q*) ranging from 0.789 (TR) to 0.997 (ST). Within the Prachub Kirikan location (PK code), most individuals are admixed. Such samples collected from PK1a, PK1b, and PK2a generally share their proportion of ancestry in the same clusters whereas PK2b samples appear to be admixed to a different cluster (Figure 3, Additional file 2: Table S2).Principle Coordinate Analysis (PCoA) demonstrates the genetic divergence of fruit fly populations in multi-dimensional space (Figure 4). This result is also consistent with STRUCTURE analyzes. The first axis accounts for 30.25% of total variation, which separates populations ST, TR, SL, and PH from the remaining populations. The second (23.08% of total variation) mainly distinguishes SK1 and SK2 from the others. The third (16.40% of total variation) distinguishes KD and JH from the rest of the populations.

**Figure 5:**
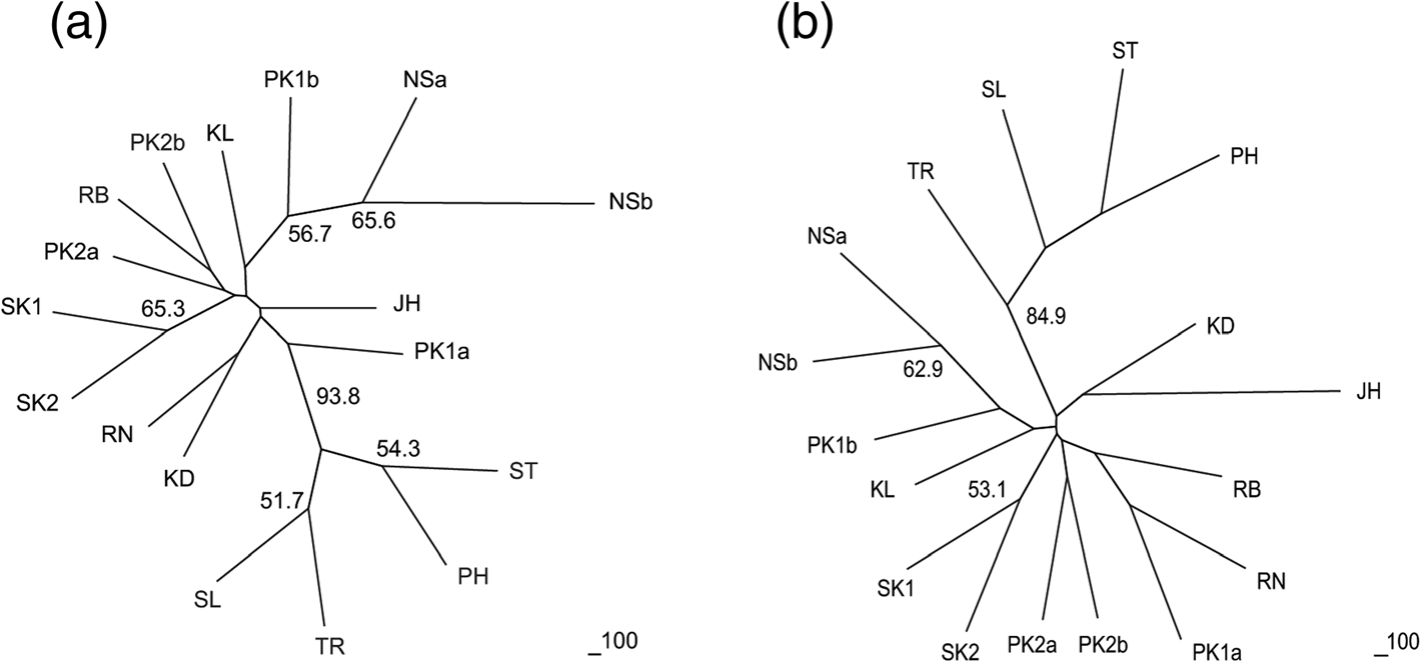
**Neighbor-joining trees based on the genetic distance derived from (a) Nei’s genetic distance**[48]**and (b) the proportion of shared alleles**[49]**.** The number at each node indicates the bootstrap percentile values after 1,000 replications.

The main feature of neighbor-joining trees, based on Nei’s genetic distance [48] and the proportion of shared alleles [49], is a clear-cut separation of four populations (ST, TR, SL, and PH) from the others. Additional neighborhoods include SK1 & SK2 and NSa & NSb (Figure 5).

The analysis of molecular variance (AMOVA) shows the extent of genetic variation in different hierarchies. The populations were grouped by three criteria: morphological forms, geographical areas, and genetic structure (Table 4). In the overall analyzes, less than 5.0% of variation is attributed to the differences among groups while approximately 90% of variation is attributable to differences within populations. Non-significant difference among groups is observed with regard to morphological forms (scenario 1: *B. dorsalis* s.s. vs. *B. papaya*e, *P* = 0.555). Likewise, geographical considerations - scenario 2 (Southern Thailand vs. West Malaysia) and scenario 3 (above vs. below the Isthmus of Kra) - reveal non-significant differences (*P* = 0.058 and *P* = 0.143, respectively). However, significant differences are observed (*P* < 0.050) when all samples were grouped by the genetic coancestry clusters at *K* = 2 or 3.

**Table 4:** Analysis of molecular variance (AMOVA) tests

	**Among groups**				**Among populations within groups**				**Within populations**			
Group^*^	***V***_**a**_	**Percentage**	***P***	***F***_**CT**_	***V***_**b**_	**Percentage**	***P***	***F***_**SC**_	***V***_**c**_	**Percentage**		***P***	***F***_**ST**_
1	−0.0082	−0.24	0.5552	−0.0024	0.3320	9.84	<0.0001	0.0982	3.0499	90.40	<0.0001	0.0960
2	0.0367	1.08	0.0577	0.0108	0.3094	9.11	<0.0001	0.0921	3.0499	89.81	<0.0001	0.1019
3	0.0188	0.56	0.1427	0.0056	0.3181	9.39	<0.0001	0.0945	3.0499	90.05	<0.0001	0.0995
4	0.1651	4.75	<0.0001	0.0475	0.2583	7.44	<0.0001	0.0781	3.0499	87.81	<0.0001	0.1219
5	0.1548	4.51	<0.0001	0.0451	0.2289	6.67	<0.0001	0.0698	3.0499	88.83	<0.0001	0.1117

^*^1: Morphological form (*B. dorsalis* s.s. vs *B. papayae*).

2: Geographical area (Southern Thailand vs West Malaysia).

3: Geographical area (above vs below Isthmus of Kra).

4: Genetic coancestry (*K* = 2).

5: Genetic coancestry (*K* = 3).

### Demographic analyzes

Recent migration: The individual assignment analysis (*m*) was performed using GENECLASS 2.0 [52] as shown in Table 5. All italic values along the diagonal of the matrix indicate the proportions of individuals derived from their source population. The highest value belongs to NSa, the smallest sample size population, (*m* = 0.888) while the lowest is RB (*m* = 0.517). The limitation of genetic sharing among populations does not depend on type of morphological form and geographical area. RN shows asymmetric migration to almost all populations, ranging between 0.103 (JH) to 0.594 (NSa), but significantly receives genetic information from only PK1a (*m* = 0.151). On the other hand, populations ST, NSa, and TR do not significantly migrate to any populations. For the first year of collection of sympatric populations, the migration rate from PK1a to PK1b is 0.283, while the opposite migration rate is only 0.131. The same situation is not evident two years later. PK2a and PK2b populations reveal similar migration rates in both directions (*m* = 0.112 and *m* = 0.145, respectively). In the case of NSa and NSb, the other sympatric populations, an asymmetric migration rate is detected from NSb to NSa (*m* = 0.611).

**Table 5:** **Assignment analysis of the 17 populations of *B. dorsalis *** **s.s. and *B. papayae *** **forms using GENECLASS**[52]

**Assignment**	**Reference populations**																
**Individuals**	**RB**	**PK1a**	**PK2a**	**RN**	**ST**	**NSa**	**PK1b**	**PK2b**	**NSb**	**SK1**	**SK2**	**KD**	**TR**	**SL**	**PH**	**KL**	**JH**
RB	***0.517***	0.088	0.067	**0.119**	0.000	0.009	0.066	0.042	0.066	0.012	0.037	0.046	0.002	0.008	0.010	0.002	0.070
PK1a	0.097	***0.554***	**0.188**	**0.280**	0.001	0.011	**0.131**	0.088	0.047	0.026	0.008	0.058	0.043	0.029	**0.110**	0.017	**0.147**
PK2a	**0.120**	**0.172**	***0.559***	**0.132**	0.001	0.016	**0.124**	**0.145**	0.044	0.072	0.023	0.038	0.042	0.054	**0.100**	0.033	**0.144**
RN	0.045	**0.151**	0.099	***0.665***	0.001	0.023	0.053	0.047	0.026	0.022	0.013	0.079	0.003	0.015	0.028	0.021	0.090
ST	0.029	**0.106**	**0.113**	**0.171**	***0.597***	0.006	0.057	0.085	0.048	**0.107**	0.021	0.022	0.072	0.092	**0.331**	0.013	**0.119**
NSa	**0.213**	**0.167**	**0.336**	**0.594**	0.000	***0.888***	**0.609**	**0.185**	**0.611**	0.027	0.009	**0.173**	0.055	0.001	**0.122**	**0.241**	0.034
PK1b	**0.118**	**0.283**	**0.149**	**0.219**	0.000	0.050	***0.814***	**0.143**	**0.175**	0.037	0.008	**0.104**	0.054	0.011	0.036	0.020	0.087
PK2b	**0.106**	**0.164**	**0.112**	**0.121**	0.000	0.034	**0.168**	***0.530***	**0.137**	0.090	0.024	0.033	0.014	0.007	0.059	0.031	**0.155**
NSb	**0.133**	**0.117**	0.067	**0.118**	0.000	0.092	**0.183**	**0.140**	***0.702***	0.015	0.024	0.069	0.008	0.001	0.048	0.030	0.016
SK1	0.057	**0.115**	**0.130**	**0.115**	0.003	0.013	**0.133**	**0.208**	0.086	***0.700***	**0.140**	0.065	0.024	0.008	0.043	0.024	**0.184**
SK2	0.068	0.036	0.034	0.052	0.000	0.005	0.017	0.037	0.060	0.075	***0.627***	0.023	0.001	0.002	0.019	0.001	0.075
KD	0.060	**0.128**	0.089	**0.238**	0.001	0.022	0.056	0.072	0.094	0.029	0.010	***0.704***	0.025	0.004	0.047	0.008	0.044
TR	0.027	**0.213**	0.058	**0.193**	0.016	0.022	0.087	0.061	0.035	0.014	0.006	0.054	***0.558***	**0.237**	**0.246**	0.029	**0.148**
SL	0.033	**0.120**	0.090	**0.121**	0.015	0.008	0.040	0.034	0.024	0.018	0.004	0.050	0.095	***0.657***	**0.189**	0.011	**0.113**
PH	0.015	0.043	0.038	0.094	0.015	0.010	0.036	0.039	0.012	0.009	0.008	0.019	0.097	**0.108**	***0.653***	0.006	0.054
KL	0.007	0.043	0.047	0.067	0.000	0.031	**0.106**	0.043	0.069	0.024	0.006	0.025	0.014	0.004	0.018	***0.586***	0.055
JH	0.013	0.037	0.042	**0.103**	0.000	0.010	0.068	0.046	0.035	0.044	0.015	0.033	0.018	0.018	0.031	0.008	***0.691***

Values of significant migration rate (*m* ≥ 0.100) are in bold. The proportions of individuals derived from the population in which they were collected in italics.

We combined the results of two programs (GENECLASS and STRUCTURE) in order to interpret congruency between genetic data and morphological forms. According to the STRUCTURE analysis, 95 individuals (approximately 25% of the total) are categorized as admixed based on their proportion of *Q* that ranges from 0.200 to 0.800 (Additional file 2: Table S2). Five populations, including ST, NSa, SK2, SL, and KL, do not contain any of these admixed individuals (Figure 3). Based on the results from the GENECLASS program, 45 of 95 individuals appear to be nonimmigrant considering the consistency between the original sampling site and the most probable population. The remaining 50 individuals are significantly classified as migrants (*m* ≥ 0.100) from at least one population. Individual numbers 10, 14, 16, 33, and 43 are potentially admixed and are also migrants from elsewhere (Additional file 2: Table S2).

Bottleneck: Under the Stepwise Mutation Model (SMM), a significant heterozygote deficit (population expansion) was observed in three populations (PK1a (*P* = 0.043), PK2b (*P* = 0.001), and SK2 (*P* = 0.003)) and no significant heterozygote excess (population bottleneck) was detected based on a two-tailed Wilcoxon signed-rank test. Using a more stringent model, the two-phase model (TPM), only two populations (PK2b (*P* = 0.0034) and SK2 (*P* = 0.0342)), still showed a recent population expansion.

Isolation by distance (IBD): All 17 *B. dorsalis* s.s. and *B. papayae* populations show no significant correlation between genetic and geographical distances [*R*^2^ = 0.056, *P* = 0.650, *F*_ST_/(1-*F*_ST_) = 0.0054 Ln (geographical distance) + (0.0703)]. When partitioning either morphological form *B. dorsalis* s.s. (*R*^2^*=* 0.008, *P* = 0.837) or morphological form *B. papayae* (*R*^2^ = 0.063, *P* = 0.173) samples, no significant IBD is confirmed. These results are similar to those from the migration analysis, indicating no limitation of gene flow across populations of morphological forms.

## Discussion

A fine-scale population genetics study of two morphological forms - *B. dorsalis* s.s. and *B. papaya*e - was carried out in areas spanning from the top of Southern Thailand to the tip of the Malay Peninsula using high-resolution microsatellite DNA markers derived from each form. It can be inferred that these two morphological forms comprise a panmictic unit. Genetic variation, population structure, and recent population demography suggest a high feasibility for area-wide integrated pest management using sterile insect technique (AW-IPM-SIT) control programs. A combination of population genetic tools and morphological characterization may be necessary to better understand target pest populations.

### Comparable intra- and inter-specific variations

The factors affecting microsatellite DNA variability include locus- and genome-specific mutation rates [56]. Microsatellite DNA markers are always isolated and characterized from a single species. In such, specific loci with high genetic variability are chosen. However, this genetic variability may not be inherently transferred when the marker system is applied to another species. If the recipient genome is very different, the level of genetic variability of the orthologues will be biased (ascertainment bias) [28-30]. In this study, we did not directly analyze the mutation rates at each microsatellite locus, but we statistically compared genetic variation using common parameters (i.e., effective number of alleles, variance in allele size range, and heterozygosity) among 12 loci for 17 populations. We found that the given microsatellite loci were highly polymorphic for all populations, although the *B. papayae*-derived loci were less variable than *B. dorsalis* s.s. for all parameters. The explanation involves several factors that influenced microsatellite mutation rate, such as repeat number, repeat type, flanking sequence (GC content), chromosome location, and base substitutions in the microsatellite arrays [57]. In our case, all microsatellite DNA loci used have similar repeat numbers, the same type of GT/CA motif (Additional file 1: Table S1) and lack data regarding chromosome location. The different level of variability between two sets of microsatellite loci may be influenced by flanking sequences and base substitution in the microsatellite motifs. The low GC content of flanking sequences may have effect on high microsatellite variability [58]. In this case, primer sequences (as flanking sequences) of *B. dorsalis* s.s.-derived loci (ranging from 0.16 to 0.55, mean = 0.40 ± 0.10) are relatively lower than *B. papayae*-derived loci (ranging from 0.46 to 0.65, mean = 0.53 ± 0.07). It is possible that *B. dorsalis* s.s.-derived loci tend to provide a higher variability of microsatellite. Likewise, motifs of *B. dorsalis* s.s.-derived loci showed relatively higher base substitution than the other. This also supports the high variability in *B. dorsalis* s.s.-derived loci. In addition, no significant genetic differences were observed among all populations, the two forms, the two countries, and also the interaction between marker derivation and species factors. Consequently, it can be deduced that the genetic polymorphisms of all tested loci may be shaped by the issue of population histories, not by ascertainment bias. Therefore, using two different species-derived sets of microsatellite loci, if available, could help us to clarify the source of genetic variability as well as to avoid biasing the data. This may help us to not undermine the determination of sympatric species that have no interbreeding between groups [4].

Studies using high-resolution microsatellite markers had been successfully used to delineate the limits of other species complexes (e.g., [5,19,59]); however, our study does not support the hypothesis that *B. dorsalis* s.s. and *B. papayae* forms are cryptic species in the investigated areas. Independent studies using different samples and sets of microsatellite markers (i.e., microsatellite-derived markers from only *B. dorsalis* s.s. [14] and a combination of microsatellite-derived markers from *B. dorsalis* s.s. & *B. papayae* (this study)) present a comparable genetic variation between two morphological forms. These facts imply that *F*_ST_ of the given species cannot be resolved. A plausible explanation is that both forms comprise a panmictic unit or recently diverged, and/or they are connected through a high level of hybridization [4]. If both species have recently diverged, intra-specific microsatellite variation should correlate with inter-specific variation as a result of incomplete lineage sorting or still maintain a low level of gene flow. We found that an observed intra-specific difference was generally comparable to an observed inter-specific divergence. Moreover, other data derived from various DNA barcodes such as COI [13], *cox*1, *nad*4-3′, CAD, *period*, ITS1, ITS2 [16], EF-1α, PER [60], and other non-morphological characteristics such as pheromone profile [61], and mating competition between both species [15], along with morphometric analyzes [13,14], provide congruent results, supporting the recent divergence hypothesis. Nonetheless, microsatellite DNA markers may provide additional insights into population genetics.

Other evidence of *F*_ST_ between two morphological forms and among populations was significant, but quite low. Approximately 2% to 18% of genetic diversity is the result of genetic differentiation among *B. dorsalis* s.s. and *B. papayae* populations. A high level of gene flow between populations or recent species divergence is the most likely explanation. Considering the genetic diversity of the single species *B. dorsalis* s.s., using at least the same seven microsatellite loci as the current study, we found that approximately one to six percent (1-6%) and up to 30% of genetic diversity are the consequence of genetic differentiation among the populations at the level of micro-geography (populations collected from Thailand) and macro-geography (covering populations collected from the hypothetical area of origin and other areas), respectively [62]. Comparing the two morphological forms and the single species *B. dorsalis* s.s., the *F*_ST_ of former falls between micro- and macro-geographical values. However, this interpretation must be viewed with caution because the *F*_ST_ value is independent of the particular characteristics of individual loci or alleles and is influenced by the geographical differences of sampling locations [63]. Finally, the AMOVA result indicated that there is no genetic heterogeneity when all samples were grouped based on morphological and geographical criteria. We can infer that the genetic variation detected from all samples belong to the population level but not the cryptic species level. For that reason, we have no strong evidence to disprove the hypothesis that both morphological forms are actually a panmictic unit [13-16,60] or very recently divergent. More sample collections from other geographical areas or different ecological niches may be required in order to generalize the status of the population genetic divergence, although other diagnosable characters have not yet evolved or been established.

### Population dynamics in allopatric and sympatric areas

We inferred population dynamics in allopatric and sympatric areas with regard to recent migration and population expansion/contraction. The narrowest part of the Thai-Malay Peninsula - Kra Isthmus - is located between Ranong and Chumphon Provinces, Thailand. Despite the fact that this area was proposed to be a biogeographic transition zone for *B. dorsalis* s.s. and *B. papayae*[8,11], our data do not indicate any genetic barrier or genetic heterogeneity of both forms across this zone (congruent with [14]). In addition, the RN population appears to be a genetic source for the morphological forms of *B. dorsalis* s.s. and *B. papayae* in our studied areas. The data shows a high level of genetic variation, a low level of genetic differentiation, and recent asymmetric migration to almost all populations. Similarly, Ratchaburi was previously proposed as a genetic source of *B. dorsalis* s.s. in Thailand when samples from Southern Thailand and West Malaysia were not taken [62]. However, Ratchaburi is such a legitimate area because there are plenty of commercial fruit orchards and distribution centers. The current study illustrates that RB has as high a genetic variation as RN but serves as a recent genetic source for a few populations in Southern Thailand. The different types of preferred hosts and climates between the south of Southern Thailand and northward [10] may be the underlying factors affecting the different population dynamics. On the other hand, ST and TR may indicate recently introduced populations. These populations have a low level of genetic variation, clearly indicated by the low number and frequency of private alleles. These populations have recently received genetic information from other populations, but not *vice versa*. The genetic ancestry of a few fruit fly samples (1.32%) cannot be traced back to any investigated populations, although nearby populations were no more than 200 km away. Adequate representative populations could be sampled in order to expand on this type of population dynamics detail, which may help the implementation of SIT programs.

Population dynamics is also illustrated using the data from two different collections at two different time points, but in the same area of Prachub Kirikan Province. Populations PK1a and PK2a, the same morphological form *B. dorsalis* s.s., seem to maintain their stable populations, which is inferred from the comparable population size and genetic variability. On the other hand, populations of the other morphological form appear to be expanding. The first collection, PK1b, has a small population size (*N* < 10); however, two years later, PK2b was observed to have increased in population size as well as in genetic variability. According to inferences made from allopatric and sympatric populations, the fruit fly population dynamics may not be at equilibrium in Southern Thailand and West Malaysia. This demonstrates that population genetic study using multiple-time-point sample collection is encouraged to infer delicate population dynamics processes.

### Feasibility of an AW-IPM-SIT program for *B. dorsalis* s.s. and *B. papayae* in Southern Thailand and West Malaysia

Fruit fly surveillance using a combination of tools (e.g., monitoring traps, species identification, and fruit sampling) is a key concept for planning and implementation of an AW-IPM-SIT program. Utilization of the traditional approach based on morphology and type of fruit fly hosts for characterization of pest species may result in misidentification. Sample collection from significantly isolated geographical areas without intermediate sites could provide samples with more discrete morphological characters than geographically closer sites [14]. Although our genetic study does not agree with the traditional morphological-based taxonomic species status of *B. dorsalis* s.s. and *B. papayae* forms, it is consistent with other independent identification approaches using non-morphological characteristics and morphometric analyzes (as mentioned before). This implies that morphological variation is not a standalone indicator of species boundaries. Extreme environments can impose stabilizing selection on morphological characters which have nothing to do with the species differentiation process. In contrast, selection of non-morphological traits such as behavior, molecular markers, or reproductive character, can accompany speciation [4]. Incorporation of data from non-morphological-based approaches (such as population genetics) may supply strong evidence that can describe species status.

The release of sterile male only is a very crucial step to improve SIT, in terms of the cost and biological efficiency in the field [2]. Development and evaluation of genetic sexing strains (GSSs), separating males from females, is still a challenge for researcher. In the tephritid fruit fly, several GSSs have been developed using classical and transgenic technology [64]. Over the last two decades, few of them have been available due to genetic instability, poor mating performance, and delayed regulatory approval, for both mass-rearing and field application. There are *Ceraitis capitata* (Vienna 8) [65], *Bactrocera dorsalis* (Salaya1) [66], and *Anastrepha ludens* (Tapachula-7) [67]. Therefore, it is very importance that one strain can be used for controlling other members of the same complex. Whether or not both of the current morphological forms are definitely defined as distinct taxa, releasing the sterile Salaya1 strain to control *B. dorsalis* s.s. and *B. papayae* is possible, in an area-wide sense, in Southern Thailand and West Malaysia. Several reliable lines of evidence (e.g., mating competitiveness field cage tests [15] and estimation of gene flow in this study) support that *B. dorsalis* s.s. and *B. papayae* forms may be a single target pest. However, mating competitiveness field cage tests need to be done for additional confirmation before actual implementation in the studied areas. A successful showcase study of released sterile *B. dorsalis* s.s. (originally derived from Hawaii) against *B. carambolae* in Suriname had been reported [68].

However, we detect ongoing delicate population dynamics processes such as population reduction/expansion and migration within studied areas. The phenomena could produce subpopulations by various scenarios and subsequently impact the effectiveness of AW-IPM-SIT. For example, new immigrants may be introduced into a new microhabitat which may form a pocket population. When the SIT activity is less intense or no longer practiced, they may be founders for an incursion scenario [69]. This reminds us that before and during the implementation of an AW-IPM-SIT program, a population genetic survey is highly recommended, especially when cryptic species and/or population isolation issues are involved.

## Conclusions

In summary, there is no status for cryptic species between two morphological forms - *B. dorsalis* s.s. and *B. papayae -* in Southern Thailand and West Malaysia based on the variations of microsatellite DNA markers derived from both species. Hence, both forms may be treated as a single target pest for an SIT control program. However, resolution of genetic isolation and morphology are not congruent in species identification. The characterization of a pest population using multiple approaches may ensure the effectiveness and feasibility of the SIT-based method.

## Competing interests

The authors declare that they have no competing interests.

## Authors’ contributions

NA and ST conceived and designed the research project; NA and SI performed the genotyping; NA conducted the population genetic analyzes; NA wrote the manuscript. All authors reviewed the results from the data analysis and approved the final manuscript.

## Supplementary Material

Additional file 1: Table S1 Description of each primer used in the current study. Click here for file

Additional file 2: Table S2 Results of migration analyses using STRUCTURE Click here for file
