# Supplementary material for: Evidence of weak genetic structure and recent gene flow between Bactrocera dorsalis s.s. and B. papayae, across Southern Thailand and West Malaysia, supporting a single target pest for SIT applications
Source: BMC Genet. 2014 Jun 14;15:70. doi: 10.1186/1471-2156-15-70 (PMC4071329; doi:10.1186/1471-2156-15-70)
Supplement: Additional file 1: Table S1 — Description of each primer used in the current study. [file 1471-2156-15-70-S1.doc]

# Additional File

Additional file 1 – Description of each primer used in the current study

| Locus | Repeat motif | Primers | *T*_a_ | Reference |
| --- | --- | --- | --- | --- |
| *Bd*1 | CT(CA)_4_CG(CA)_2_ | F: TGCTTAACAGTAATTGCTCC | 59 | [31] |
|  |  | R: TAAGCAGTAAACAATAAAGTTC |  |  |
| *Bd*9 | GA(GT)_7_GA | F: GCTGATATGTGTGCGTCTTA | 62 | [31] |
|  |  | R: ATCTCGTATTGTGGTTGCTT |  |  |
| *Bd*15 | (CA)_8_CGCAA(CA)_4_CGTG(TACA)_3_ | F: TGCCTTGTGCTATTTAATC | 55 | [31] |
|  |  | R: AAATAAACAAAACAAAATG |  |  |
| *Bd*19 | (TATG)_2_(TA)_2_(TG)_6_AG(TG)_2_ | F: TAGATGGAGATGGGTGCGTG | 67 | [31] |
|  |  | R: GCGTGTTCACAAGGACTAATC |  |  |
| *Bd*39 | (GT)_8_ | F: GGTCAAACAAATCACTCAG | 60 | [31] |
|  |  | R: CCGTTATATCAGGCAAATC |  |  |
| *Bd*42 | (CA)_7_(TA)_7_TG(TA)_2_GC(CA)_3_TA | F: GCACAGTGAGCGTTACAAG | 62 | [31] |
|  |  | R: ACACAAAAAACGCTCAAGTC |  |  |
| *Bd*85B | (CA)_6_CCCACC | F: ACTTCATTACACTTTCGTTG | 59 | [31] |
|  |  | R: GCTGGAATGAAAAGTCTC |  |  |
| *Bp*58 | (TG)_8_(CG)_2_ | F: TGAGCAGTATACATGCATACTCGC | 54 | [18] |
|  |  | R: AGTGCATTTGCCCAGCAGGTTG |  |  |
| *Bp*73 | (GT)_5_ | F: AGCGAAAACCAACTACTACCG | 54 | [18] |
|  |  | R: CCACTACTTCATCTTGTTCCTG |  |  |
| *Bp*125 | (TG)_6_CG(TG) | F: ATGTCAGCTGTAAGCGCAACTG | 54 | [18] |
|  |  | R: TTCGCCGTTAAATGCGCCAC |  |  |
| *Bp*173 | (TG)_5_(CGTG)_2_ | F: CACGTCCTAACGCCGACCAG | 54 | [18] |
|  |  | R: GAGCGCCCAAGCATGTCAACG |  |  |
| *Bp*181 | (AC)_8_ | F: AAAGGTGCATGCCTTCGTGTAG | 54 | [18] |
|  |  | R: TGGCTTCATCGATGAATCTGCG |  |  |
