# Supplementary material for: Evidence of weak genetic structure and recent gene flow between Bactrocera dorsalis s.s. and B. papayae, across Southern Thailand and West Malaysia, supporting a single target pest for SIT applications
Source: BMC Genet. 2014 Jun 14;15:70. doi: 10.1186/1471-2156-15-70 (PMC4071329; doi:10.1186/1471-2156-15-70)
Supplement: Additional file 2: Table S2 — Results of migration analyses using STRUCTURE [file 1471-2156-15-70-S2.doc]

# Additional File

Additional file 2 - Results of migration analyses using STRUCTURE [40-41] and GENECLASS [52]

|  | Original | Assumed species | Hypothetical genetic cluster^**^ | | | GENECLASS test (at *P* = 0.01)^***^ | | Assumed species |
| --- | --- | --- | --- | --- | --- | --- | --- | --- |
| No. | code | based on morphological form^*^ [7] | 1 | 2 | 3 | Most probable population | Second rank population | based on GENECLASS^*^ |
| 1 | RB_03 | Bd | 0.015 | **0.746** | **0.239** | RB (**0.193**) | KD (**0.188**) | Bd/Bp |
| 2 | RB_04 | Bd | 0.003 | **0.292** | **0.705** | JH (**0.840**) | RB (**0.640**) | Bp/Bd |
| 3 | RB_06 | Bd | 0.002 | **0.320** | **0.679** | RB (**0.391**) | NSb (**0.278**) | Bd/Bp |
| 4 | RB_08 | Bd | 0.001 | **0.296** | **0.703** | RB (**0.337**) | RN (0.059) | Bd/Bd |
| 5 | RB_09 | Bd | 0.014 | **0.570** | **0.417** | RN (0.054) | RB(0.022) | Bd/Bd |
| 6 | RB_12 | Bd | 0.002 | **0.632** | **0.366** | RB (**0.699**) | JH (**0.480**) | Bd/Bp |
| 7 | RB_13 | Bd | 0.004 | **0.622** | **0.373** | RN (**0.350**) | PK2a (**0.143**) | Bd/Bd |
| 8 | RB_14 | Bd | 0.007 | **0.543** | **0.451** | JH (**0.192**) | RN (**0.121**) | Bp/Bd |
| 9 | RB_18 | Bd | 0.007 | **0.280** | **0.713** | RB (**0.563**) | NSb (**0.128**) | Bd/Bp |
| 10 | RB_24 | Bd | 0.003 | **0.583** | **0.414** | RB (0.050) | na | Bd |
| 11 | RB_25 | Bd | 0.113 | **0.702** | **0.185** | RB (**0.158**) | RN (**0.150**) | Bd/Bd |
| 12 | RB_26 | Bd | 0.005 | **0.691** | **0.304** | RB (**0.218**) | RN (**0.154**) | Bd/Bd |
| 13 | RB_27 | Bd | 0.006 | **0.748** | **0.246** | PK1b (**0.167**) | PK1a (0.052) | Bd/Bd |
| 14 | RB_28 | Bd | 0.005 | **0.201** | **0.794** | na | na | na |
| 15 | PK1a_01 | Bd | **0.537** | **0.448** | 0.015 | PK1a (**0.211**) | SL (**0.140**) | Bd/Bp |
| 16 | PK1a_02 | Bd | **0.580** | **0.399** | 0.022 | SL (0.039) | na | Bp |
| 17 | PK1a_04 | Bd | **0.319** | **0.653** | 0.028 | PK1a (**0.210**) | RN (**0.189**) | Bd/Bd |
| 18 | PK1a_06 | Bd | **0.281** | **0.606** | **0.114** | PK1a (**0.565**) | PK2b (**0.204**) | Bd/Bp |
| 19 | PK1a_10 | Bd | **0.265** | **0.711** | 0.024 | PK1a (**0.572**) | RN (**0.539**) | Bd/Bd |
| 20 | PK1a_13 | Bd | **0.446** | **0.534** | 0.020 | PH (**0.604**) | RN (**0.340**) | Bp/Bd |
| 21 | PK1a_15 | Bd | **0.265** | **0.688** | 0.047 | PK1a (**0.478**) | PK2a (**0.261**) | Bd/Bd |

^*^Bd and Bp designate *B. dorsalis* s.s. and *B. papayae*, respectively.

^**^Admixed individuals that have a proportion of shared genetic clusters between 0.200 and 0.800 are in bold.

^***^Parameter values for the simulation included 10,000 simulated individuals and the Bayesian method. Migration rates (*m* > 0.100) are in bold.

“na” indicates low migration rate (*m* <0.010), inferring potentially admixed individual and/or migrant from an unsampled population.

Additional file 1 - Results of migration analyses using STRUCTURE [40-41] and GENECLASS [52] (continued)

|  | Original | Assumed species | Hypothetical genetic cluster^**^ | | | GENECLASS test (at *P* = 0.01)^***^ | | Assumed species |
| --- | --- | --- | --- | --- | --- | --- | --- | --- |
| No. | code | based on morphological form^*^ [7] | 1 | 2 | 3 | Most probable population | Second rank population | based on GENECLASS^*^ |
| 22 | PK1a_17 | Bd | **0.228** | **0.719** | 0.053 | PK1a (0.035) | JH (0.030) | Bd/Bp |
| 23 | PK1a_19 | Bd | **0.296** | **0.687** | 0.018 | PK2a (**0.820**) | RN (**0.793**) | Bd/Bd |
| 24 | PK1a_20 | Bd | **0.280** | **0.699** | 0.021 | RN (**0.414**) | PK1a (**0.180**) | Bd/Bd |
| 25 | PK1a_21 | Bd | **0.556** | **0.424** | 0.020 | PK1a (**0.429**) | RN (**0.387**) | Bd/Bd |
| 26 | PK1a_22 | Bd | **0.691** | **0.286** | 0.024 | PH (0.065) | RN (0.020) | Bp/Bd |
| 27 | PK1a_23 | Bd | **0.563** | **0.417** | 0.021 | PK1a (**0.313**) | PK2b (0.046) | Bd/Bp |
| 28 | PK1a_24 | Bd | **0.631** | **0.351** | 0.019 | PH (**0.440**) | PK1a (**0.154**) | Bp/Bd |
| 29 | PK1a_25 | Bd | **0.357** | **0.269** | **0.374** | PK1b (0.050) | SK2 (0.015) | Bp/Bp |
| 30 | PK1a_26 | Bd | **0.312** | **0.627** | 0.061 | PK1a (**0.165**) | PK1b (0.015) | Bd/Bp |
| 31 | PK1a_27 | Bd | **0.377** | **0.549** | 0.074 | PK1a (0.092) | PK1b (0.064) | Bd/Bp |
| 32 | PK1a_28 | Bd | **0.321** | **0.602** | 0.077 | JH (**0.108**) | PK1a (0.099) | Bp/Bd |
| 33 | PK1a_29 | Bd | **0.563** | **0.320** | **0.117** | na | na | na |
| 34 | PK1a_30 | Bd | **0.324** | **0.647** | 0.029 | PK1a (**0.276**) | RB (**0.141**) | Bd/Bd |
| 35 | PK1b_02 | Bp | **0.194** | **0.775** | 0.031 | RN (**0.182**) | PK1a (**0.124**) | Bd/Bd |
| 36 | PK2a_10 | Bd | **0.252** | **0.710** | 0.038 | PK2a (**0.198**) | PK1a (**0.168**) | Bd/Bd |
| 37 | PK2a_12 | Bd | **0.116** | **0.592** | **0.292** | PK2a (**0.239**) | PK2b (**0.212**) | Bd/Bp |
| 38 | PK2a_13 | Bd | **0.617** | **0.335** | 0.049 | PH (**0.532**) | PK2a (**0.407**) | Bp/Bd |
| 39 | PK2a_21 | Bd | **0.695** | **0.248** | 0.057 | PH (**0.186**) | NSb (0.023) | Bp/Bp |
| 40 | PK2a_26 | Bd | **0.264** | **0.547** | **0.188** | PH (0.075) | RN (0.056) | BpBd |
| 41 | PK2a_30 | Bd | **0.709** | **0.126** | **0.165** | PK2b (**0.293**) | PH (**0.224**) | Bp/Bp |
| 42 | PK2b_06 | Bp | 0.004 | **0.241** | **0.755** | PK2a (0.031) | PK2b (0.015) | Bd/Bp |
| 43 | PK2b_07 | Bp | 0.022 | **0.789** | **0.188** | NSb (0.017) | na | Bp |

^*^Bd and Bp designate *B. dorsalis* s.s. and *B. papayae*, respectively.

^**^Admixed individuals that have a proportion of shared genetic clusters between 0.200 and 0.800 are in bold.

^***^Parameter values for the simulation included 10,000 simulated individuals and Bayesian method. Migration rates (*m* > 0.100) are in bold.

“na” indicates low migration rate (*m* <0.010), inferring the potentially admixed and/or migrant from an unsampled population.

Additional file 1 - Results of migration analyses using STRUCTURE [40-41] and GENECLASS [52] (continued)

|  | Original | Assumed species | Hypothetical genetic cluster^**^ | | | GENECLASS test (at *P* = 0.01)^***^ | | Assumed species |
| --- | --- | --- | --- | --- | --- | --- | --- | --- |
| No. | code | based on morphological form^*^ [7] | 1 | 2 | 3 | Most probable population | Second rank population | based on GENECLASS^*^ |
| 44 | PK2b_08 | Bp | 0.066 | **0.604** | **0.330** | PK2b (**0.590**) | NSb (**0.486**) | Bp/Bp |
| 45 | PK2b_10 | Bp | 0.005 | **0.647** | **0.348** | PK2b (**0.140**) | PK1b (0.064) | Bp/Bp |
| 46 | PK2b_12 | Bp | 0.004 | **0.765** | **0.231** | PK2b (**0.337**) | NSb (0.055) | Bp/Bp |
| 47 | PK2b_15 | Bp | 0.012 | **0.512** | **0.476** | NSb (**0.135**) | PK2b (**0.127**) | Bp/Bp |
| 48 | PK2b_17 | Bp | 0.015 | **0.771** | **0.214** | RN (**0.351**) | PH (**0.326**) | Bd/Bp |
| 49 | PK2b_22 | Bp | 0.012 | **0.403** | **0.586** | SK1 (**0.125**) | PK2b (0.098) | Bp/Bp |
| 50 | PK2b_24 | Bp | 0.008 | **0.760** | **0.232** | PK2b (**0.869**) | NSb (**0.852**) | Bp/Bp |
| 51 | PK2b_25 | Bp | 0.004 | **0.715** | **0.281** | PK1b (**0.163**) | PK2a (0.041) | Bp/Bd |
| 52 | PK2b_26 | Bp | 0.004 | **0.373** | **0.623** | SK1 (**0.321**) | SK2 (0.052) | Bp/Bp |
| 53 | RN_06 | Bd | **0.206** | **0.780** | 0.014 | RN (**0.211**) | PK1a (**0.168**) | Bd/Bd |
| 54 | RN_11 | Bd | **0.508** | **0.486** | 0.006 | RN (0.072) | PH (0.044) | Bd/Bp |
| 55 | RN_12 | Bd | **0.507** | **0.485** | 0.008 | RN (0.088) | PH (0.046) | Bd/Bp |
| 56 | RN_13 | Bd | **0.211** | **0.774** | 0.016 | RN (**0.196**) | PK2b (**0.111**) | Bd/Bp |
| 57 | RN_21 | Bd | **0.363** | **0.624** | 0.013 | RN (**0.592**) | PK1a (**0.389**) | Bd/Bd |
| 58 | NSb_01 | Bp | 0.028 | **0.609** | **0.363** | RB (**0.514**) | NSb (**0.480**) | Bd/Bp |
| 59 | NSb_02 | Bp | 0.082 | **0.698** | **0.220** | RN (**0.485**) | PK1b (**0.395**) | Bd/Bp |
| 60 | NSb_03 | Bp | 0.032 | **0.646** | **0.323** | NSb (**0.659**) | RN (**0.277**) | Bp/Bd |
| 61 | NSb_04 | Bp | 0.065 | **0.407** | **0.524** | PK2b (0.080) | SK2 (0.080) | Bp/Bp |
| 62 | NSb_05 | Bp | 0.028 | **0.524** | **0.448** | NSb (**0.397**) | PK2b (**0.382**) | B/Bp |
| 63 | NSb_06 | Bp | 0.028 | **0.541** | **0.431** | NSb (**0.406**) | PK2b (**0.334**) | Bp/Bp |
| 64 | NSb_07 | Bp | 0.036 | **0.480** | **0.485** | NSb (**0.129**) | PK2b (**0.103**) | Bp/Bp |

^*^Bd and Bp designate *B. dorsalis* s.s. and *B. papayae*, respectively.

^**^Admixed individuals are in bold which have the proportion of shared genetic clusters between 0.200 to 0.800.

^***^Parameter values for the simulation included 10,000 simulated individuals and Bayesian method. Migration rates (*m* > 0.100) are in bold.

“na” indicates low migration rate (*m* <0.010), inferring the potentially admixed and/or migrant from an unsampled population.

Additional file 1 - Results of migration analyses using STRUCTURE [40-41] and GENECLASS [52] (continued)

|  | Original | Assumed species | Hypothetical genetic cluster^**^ | | | GENECLASS test (at *P* = 0.01)^***^ | | Assumed species |
| --- | --- | --- | --- | --- | --- | --- | --- | --- |
| No. | code | based on morphological form^*^ [7] | 1 | 2 | 3 | Most probable population | Second rank population | based on GENECLASS^*^ |
| 65 | NSb_08 | Bp | 0.046 | **0.508** | **0.447** | RB (**0.179**) | RN (**0.111**) | Bd/Bd |
| 66 | NSb_09 | Bp | 0.021 | **0.703** | **0.277** | PK2b (**0.134**) | PK1a (0.088) | Bp/Ba |
| 67 | NSb_10 | Bp | 0.026 | **0.426** | **0.548** | NSb (0.050) | SK2 (0.027) | Bp/Bp |
| 68 | NSb_11 | Bp | 0.029 | **0.700** | **0.271** | NSb (**0.609**) | KD (**0.456**) | Bp/Bp |
| 69 | NSb_12 | Bp | **0.106** | **0.614** | **0.280** | RN (**0.238**) | PK1b (**0.153**) | Bd/Bd |
| 70 | SK1_01 | Bp | **0.110** | **0.102** | **0.788** | SK2 (0.018) | SK1 (0.017) | Bp/Bp |
| 71 | SK1_02 | Bp | **0.109** | **0.133** | **0.758** | SK1 (**0.119**) | PK2b (0.082) | Bp/Bp |
| 72 | SK1_03 | Bp | 0.071 | **0.314** | **0.615** | SK2 (**0.202**) | RB (0.097) | Bp/Bd |
| 73 | SK1_04 | Bp | **0.128** | **0.292** | **0.581** | SK2 (**0.403**) | PK2b (**0.363**) | Bp/Bp |
| 74 | SK1_05 | Bp | 0.088 | **0.178** | **0.734** | SK2 (**0.162**) | PK2a (0.099) | Bp/Bd |
| 75 | SK1_06 | Bp | **0.206** | **0.354** | **0.439** | JH (**0.410**) | SK1 (0.031) | Bp/Bp |
| 76 | SK1_07 | Bp | **0.325** | **0.170** | **0.505** | SK1 (**0.637**) | JH (**0.282**) | Bp/Bp |
| 77 | SK1_08 | Bp | **0.312** | **0.193** | **0.495** | SK1 (**0.524**) | RN (**0.150**) | Bp/Bd |
| 78 | SK1_09 | Bp | **0.123** | **0.208** | **0.669** | SK1 (**0.835**) | JH (**0.563**) | Bp/Bp |
| 79 | SK1_10 | Bp | **0.227** | **0.234** | **0.539** | SK1 (0.079) | JH (0.067) | Bp/Bp |
| 80 | SK1_11 | Bp | **0.299** | **0.164** | **0.537** | SK1 (**0.638**) | PK2b (**0.381**) | BpBp |
| 81 | SK1_12 | Bp | **0.177** | **0.265** | **0.558** | PK2a (**0.315**) | RN (**0.299**) | Bd/Bd |
| 82 | SK1_13 | Bp | **0.132** | **0.183** | **0.685** | SK1 (**0.783**) | PK2b (**0.730**) | Bp/Bp |
| 83 | SK1_14 | Bp | **0.190** | **0.328** | **0.482** | SK1 (**0.614**) | PK1b (**0.516**) | Bp/Bp |
| 84 | SK1_15 | Bp | **0.272** | **0.209** | **0.519** | SK1 (**0.892**) | JH (**0.699**) | Bp/Bp |
| 85 | SK1_16 | Bp | **0.163** | **0.424** | **0.413** | PK1b (**0.226**) | SK1 (**0.109**) | Bp/Bp |

^*^Bd and Bp designate *B. dorsalis* s.s. and *B. papayae*, respectively.

^**^Admixed individuals are in bold which have the proportion of shared genetic clusters between 0.200 to 0.800.

^***^Parameter values for the simulation included 10,000 simulated individuals and Bayesian method. Migration rates (*m* > 0.100) are in bold.

“na” indicates low migration rate (*m* <0.010), inferring the potentially admixed and/or migrant from an unsampled population.

Additional file 1 – Results of migration analyses using STRUCTURE [40-41] and GENECLASS [52] analyses. (continued)

|  | Original | Assumed species | Hypothetical genetic cluster^**^ | | | GENECLASS test (at *P* = 0.01)^***^ | | Assumed species |
| --- | --- | --- | --- | --- | --- | --- | --- | --- |
| No. | code | based on morphological form^*^ [7] | 1 | 2 | 3 | Most probable population | Second rank population | based on GENECLASS^*^ |
| 86 | KD_18 | Bp | 0.011 | **0.789** | **0.200** | NSb (0.030) | PK1b (0.022) | Bp/Bp |
| 87 | TR_10 | Bp | **0.431** | **0.565** | 0.004 | PK2b (**0.807**) | PK1b (**0.800**) | Bp/Bp |
| 88 | TR_14 | Bp | **0.681** | **0.311** | 0.008 | PK1a (0.049) | KD (0.041) | Bd/Bp |
| 89 | TR_23 | Bp | **0.771** | **0.214** | 0.015 | JH (**0.252**) | TR (**0.239**) | Bp/Bp |
| 90 | TR_27 | Bp | **0.725** | **0.269** | 0.007 | PK1b (**0.271**) | PK1a (**0.218**) | Bp/Bd |
| 91 | PH_03 | Bp | **0.503** | 0.020 | **0.477** | SK2 (0.012) | na | Bp |
| 92 | JH_13 | Bp | **0.171** | **0.797** | 0.033 | PK1b (0.079) | JH (0.072) | Bp/Bp |
| 93 | JH_14 | Bp | **0.354** | **0.619** | 0.027 | RN (**0.479**) | PK2a (0.220) | Bd/Bd |
| 94 | JH_16 | Bp | **0.292** | **0.638** | 0.070 | PH (**0.135**) | SL (0.022) | Bp/Bp |
| 95 | JH_17 | Bp | **0.245** | **0.672** | 0.083 | PK2a (0.050) | SL (0.017) | Bd/Bp |

^*^Bd and Bp designate *B. dorsalis* s.s. and *B. papayae*, respectively.

^**^Admixed individuals are in bold which have the proportion of shared genetic clusters between 0.200 to 0.800.

^***^Parameter values for the simulation included 10,000 simulated individuals and Bayesian method. Migration rates (*m* > 0.100) are in bold.

“na” indicates low migration rate (*m* <0.010), inferring the potentially admixed and/or migrant from an unsampled population.
